# The prevalence of headache disorders in children and adolescents in Mongolia: a nationwide schools-based study

**DOI:** 10.1186/s10194-020-01175-6

**Published:** 2020-09-01

**Authors:** Otgonbayar Luvsannorov, Tsengunmaa Anisbayar, Munkhzul Davaasuren, Otgonzaya Baatar, Khaliunaa Batmagnai, Khulan Tumurbaatar, Sarantuya Enkhbaatar, Derya Uluduz, Tayyar Şaşmaz, Elif Tuğçe Solmaz, Timothy J. Steiner

**Affiliations:** 1 Department of Neurology, Mongolian National University of Medical Sciences, Ulaanbaatar, Mongolia; 2 Neurology Department, Cerrahpaşa School of Medicine, Istanbul University, Istanbul, Turkey; 3 Public Health Department, School of Medicine, Mersin University, Mersin, Turkey; 4 Department of Neuromedicine and Movement Science, Norwegian University of Science and Technology, Edvard Griegs gate, Trondheim, Norway; 5 Division of Brain Sciences, Imperial College London, London, UK

**Keywords:** Child and adolescent headache, Migraine, Tension-type headache, Medication-overuse headache, Undifferentiated headache, Epidemiology, Prevalence, Schools-based study, Mongolia, Global campaign against headache

## Abstract

**Background:**

The Global Campaign against Headache collects data from children (7–11 years) and adolescents (12–17 years) both to inform health and education policies and to contribute to the Global Burden of Disease (GBD) study. This survey in Mongolia was part of this global enquiry.

**Methods:**

Following the generic protocol for the global enquiry, this was a schools-based cross-sectional survey. Self-completed structured questionnaires were administered, within classes, in seven schools in four districts of the Capital city and three rural areas of Mongolia, selected to represent the country’s diversities. Headache diagnostic questions were based on ICHD-3 criteria but for the inclusion of undifferentiated headache (UdH).

**Results:**

Of 4515 potential participants, 4266 completed the questionnaire (children 2241 [52.5%], adolescents 2025 [47.5%]; males 2107 [49.4%], females 2159 [50.6%]). Children were therefore slightly over-represented, although overall mean age was 11.3 ± 3.3 years (range: 6–17; median 11). The non-participation proportion was 4.5%. Observed lifetime prevalence of headache was 81.0%. Gender- and age-adjusted 1-year prevalence was 59.4% (migraine: 27.3%; tension-type headache [TTH]: 16.1%; UdH: 6.6%; all headache on ≥15 days/month: 4.2%; probable medication-overuse headache: 0.7%). All headache types except UdH were more prevalent among females than males, and all were more prevalent among adolescents than children, although UdH represented a higher proportion of all headache in children (13.0%) than in adolescents (10.0%). Headache yesterday was reported by 15.9% of the sample, 26.0% of those with headache.

**Conclusions:**

At least in adolescents, headache in Mongolia is no less common than in adults. The clear difference from similar studies in other countries was a lower prevalence of UdH, perhaps a consequence of reporting bias in a non-troublesome headache (mild and short-lasting by definition). This study informs policy in Mongolia and, with no similar study yet from elsewhere in Western Pacific Region, makes an important contribution to the global enquiry.

## Introduction

While headache disorders collectively are clearly established as the second highest cause of disability worldwide [1–6], the prevalence estimates on which this is based depend very largely on adult studies, including the many population-based surveys supported by *Lifting The Burden* (LTB) as part of The Global Campaign against Headache [7–10]. Studies in children and adolescents have been relatively few, with the prevalence of headache disorders much less well measured. LTB, in collaboration with the International Headache Society (IHS), is now undertaking a global programme of schools-based studies using a standardised protocol [11] to collect data from children aged 6–11 years and adolescents aged 12–17 years in countries in all world regions.

This study in Mongolia, the first in Western Pacific Region, is conducted as part of this global enquiry. Although a recent survey supported by LTB found high levels of adult headache in this country [12], nothing is currently known here of headache in children and adolescents, who make up one third of the country’s population. Schooling is mandatory between ages 6 and 17 years in Mongolia, with 99% of children completing primary school and high net attendance at secondary school (urban 97%, rural 89%). Schools-based enquiry is therefore a valid means of data collection here.

The study focuses on the headache disorders with public-health importance. In adults, the principal contributors to headache-attributed burden are migraine, tension-type headache (TTH) and headache occurring on ≥15 days/month including medication-overuse headache (MOH) [1–6]. In children and adolescents, similar studies in Turkey [13], Lithuania [14] and Ethiopia [15] identified another prevalent headache disorder (undifferentiated headache [UdH]) characterised by mild pain of short duration (< 1 h) [11, 12], not diagnosable as migraine or TTH but believed to be expressions of these by the immature brain [13].

As well as adding to knowledge and understanding of the global burden of headache, an important purpose of this study is to inform local health and educational policies. This first paper reports prevalence; a second will report on burden.

## Methods

We followed the generic protocol for the global programme [11], conducting a cross-sectional survey in schools selected to be nationally representative. A structured questionnaire was completed by pupils under supervision within their school classes. Additional questionnaires completed by their teachers provided information characterising the schools.

### Ethics and approvals

Approval was obtained from the research ethics committee of Mongolian National University of Medical Sciences (MNUMS).

School managers and teachers at each selected school were asked, and agreed, to participate. Information sheets describing the nature and purposes of the survey were distributed to pupils in the participating schools to take home to parents or guardians. Prior verbal consent was obtained from each participating child or adolescent in accordance with the terms of ethics approval.

Data were collected anonymously. Data protection legislation was complied with.

### Sampling and recruitment

We conducted the study during a single academic term, avoiding examination periods, in 2018.

We surveyed one school in each of seven districts of the country, selected purposively to capture its socioeconomic diversity: four urban districts in the Capital, Ulaanbaatar city (Sukhbaatar, Bayngol, Songini-Khairkhan and Khan–Uul), and three rural districts outside Ulaanbaatar (Khuvsgul, Tuv and Umnugobi aimaks). While Mongolia’s urban/rural divide is estimated at 68.5:31.5 [16], we deliberately oversampled rural districts to enable comparative estimates. We ensured representativeness, as far as possible, having regard to types of school: government, private and mission schools. Within each school, we surveyed as many entire classes as were needed to include sufficient numbers spread evenly across the whole of the age-ranges 6–11 years and/or 12–17 years. Within each class, we included all children within either of these age ranges except those who, for any reason, declined to take part (or for whom parental objection was registered) or were unable to take part. We counted these as non-participants. Pupils who were absent from school on the survey day were not counted as non-participants since they were not available for inclusion.

### Numbers

In published recommendations, samples > 2000 are not justified, with limited potential gain from the necessarily increased investment of resources [17]. We aimed for 350 evaluable participants of each age 6–17 years (expected totals of 2100 children and 2100 adolescents), drawn approximately equally from all schools.

### Survey instruments

We used the child and adolescent versions of LTB’s Headache-Attributed Restriction, Disability, Social Handicap and Impaired Participation (HARDSHIP) questionnaire [11]. These instruments incorporate demographic enquiry, headache screening and diagnostic questions based on ICHD-3 criteria [18], and enquiries into headache-attributed burden, with enquiry timeframes of the preceding four weeks and one week (except for questions asking specifically about headache yesterday [HY]). The survey was mediated by an investigator and/or class teacher, who administered the questionnaires to the pupils in their classes. Pupils completed these anonymously, but with assistance, especially for younger children, when necessary.

Additional questionnaires, also developed by LTB [11], were addressed to teachers at each school enquiring into relevant school variables.

We translated all of these into Mongolian language following LTB’s translation protocol for lay documents [19].

### Data management and entry

Completed questionnaires were securely stored at MNUMS. As a quality-control measure, we performed independent double data-entry into an electronic database, with reconciliation of discrepancies by comparison with source data.

### Analysis

Analyses were performed at University of Mersin, Turkey, following the plan of previous studies [14, 15].

We made diagnoses during analysis, following the HARDSHIP algorithm [11, 20]. We first identified cases of headache reported on ≥14 days in the preceding 4 weeks, which we assumed represented headache on ≥15 days/month. We categorised these according to reported medication use in the preceding 4 weeks: probable MOH (pMOH) when on ≥14 days or, otherwise, as “other headache on ≥15 days/month”. Then the algorithm applied criteria for UdH (mild intensity and usual duration < 1 h [11]) followed by the ICHD-3 criteria [18] for definite migraine, definite TTH, probable migraine and probable TTH, in that order [20]. Remaining cases were unclassified.

We categorized schools by their locality (urban, semi-rural or rural), by pupils’ home income (teachers’ estimates of the proportions of pupils coming from low-income homes: less than one quarter [“higher income”], one quarter to one half [“middle income”] or more than half [“lower income”]), and by home proximity (teachers’ estimates of the proportions of pupils travelling for ≥1 h/day to attend: less than one quarter [“mostly close”] or more than one quarter [“many distant”]).

We used descriptive statistics: means and standard deviations (SDs) for continuous variables, and proportions (%) with 95% confidence intervals (CIs) for categorical data. We used chi-squared to evaluate differences between groups.

We estimated prevalence of each headache type, adjusting the observed values for gender and age using official population statistics for Mongolia [21]. In these analyses, we combined definite and probable migraine and definite and probable TTH [20]. We used bivariate analysis and odds ratios (ORs) to discover associations with demographic variables, then the binary logistic regression model to calculate adjusted ORs (AORs), entering gender, age group, school locality and pupils’ home income category. For comparison with the proportions reporting HY, we predicted 1-day prevalence of any headache and of each headache type from mean headache frequencies reported in response to two questions: “On how many days in the last week did you have a headache?” and “On how many days in the last 4 weeks did you have a headache?”

In all analyses, we considered *p* < 0.05 to indicate significance.

## Results

The population size according to class registers was 4744 (male 2333 [49.2%], female 2411 [50.8%]), but 229 pupils were absent on the day. Of the 4515 potential participants, 117 did not take part (57 because of illness, 60 for other, unrecorded reasons) and 132 were excluded because of incomplete responses. Therefore, the surveyed sample were *N* = 4266 (male 2107 [49.4%], female 2159 [50.6%]), with a participation proportion of 94.5%. Children (*n* = 2241 [52.5%]) were slightly oversampled in comparison with adolescents (*n* = 2025 [47.5%]). Overall mean age was 11.3 ± 3.3 years (range: 6–17; median 11).

School variables are shown in Table 1. Locality, because of our rural oversampling, did not perfectly reflect Mongolia’s estimated urban/rural divide (68.5:31.5 [16]). One fifth (20.5%) of participating pupils attended semi-rural schools, which were also those rated lower income, as defined above. A substantial minority of pupils (41.0%) were at schools where more than 25% were estimated to travel for > 1 h/day.

**Table 1:** School variables (from teachers’ questionnaires), and numbers of participant pupils affected (*N* = 4266)

Variable	Pupils affected	
	n	%
School locality		
urban	1831	42.9
semi-rural	873	20.5
rural	1562	36.6
Pupils’ home-income*		
“higher”	1378	32.3
“middle”	2015	47.2
“lower”	873	20.5
Home proximity*		
“mostly close”	2519	59.0
“many distant”	1747	41.0

*see text for explanation

### Headache

Headache ever was reported by 3456 participants (observed lifetime prevalence: 81.0%; 95% CI: 79.8–82.2%). Headache in the preceding year was reported by considerably fewer: 2617 (observed 1-year prevalence: 61.3%; adjusted for gender and age: 59.4%) (Table 2). Table 2 also shows observed and adjusted estimates for each headache type. Migraine was the most common (observed 1-year prevalence: 28.0% [definite 6.9%, probable 21.1%]; adjusted 27.3%), followed by TTH (observed 16.8% [definite 7.1%, probable 9.7%]; adjusted 16.1%), then UdH (observed 6.8%; adjusted 6.6%). Headache on ≥15 days/month was not uncommon (adjusted 4.2%), but only 0.7% were pMOH. There were 70 cases (1.6%) remaining unclassified.

**Table 2:** Crude (observed) 1-year prevalences (% [95% CIs]) of all headache and each headache type, overall and according to demographic variables, and gender- and age-adjusted prevalences (*N* = 4266)

	All headache (*n* = 2617)	Migraine (*n* = 1195)	TTH (*n* = 719)	pMOH (n = 30)	Other headache on ≥ 15 d/m (n = 157)	UdH (*n* = 290)
**Observed prevalences** (% [95% CI])						
Overall (N = 4266)	61.3 [59.8–62.8]	28.0 [26.7–29.4]	16.9 [15.8–18.0]	0.7 [0.5–1.0]	3.7 [3.1–4.3]	6.8 [6.0–7.6]
Gender						
male (*n* = 2107	56.1 [54.0–58.2]	24.8 [23.0–26.6]	15.8 [14.2–17.4]	0.5 [0.2–0.8]	2.5 [1.8–3.2]	7.0 [5.9–8.1]
female (*n* = 2159)	66.5 [64.5–68.5]	31.1 [29.2–33.1]	17.9 [16.3–19.5]	0.9 [0.5–1.3]	4.8 [3.9–5.7]	6.6 [5.6–7.7]
Age group (years)						
6–11 (n = 2241)	43.8 [41.8–45.9]	22.0 [20.3–23.7]	10.1 [8.9–11.4]	0.6 [0.3–0.9]	1.7 [1.2–2.2]	5.7 [4.7–6.7]
12–17 (n = 2025)	80.7 [79.0–82.4]	34.6 [32.5–36.7]	24.3 [22.4–26.2]	0.8 [0.4–1.2]	5.9 [4.9–6.9]	8.0 (6.8–9.1]
Pupils’ home income*						
“higher” (*n* = 1378)	62.8 [60.3–65.4]	25.2 [22.9–27.5]	19.2 [17.1–21.3]	0.5 [0.1–0.9]	3.6 [2.6–4.6]	8.1 [6.7–9.5]
“middle” (*n* = 2015)	60.1 [58.0–62.2]	28.8 [26.8–30.8]	15.8 [14.2–17.4]	0.7 [0.3–1.1]	3.8 [3.0–4.6]	6.2 [5.2–7.3]
“lower” (*n* = 873)	61.7 [58.5–64.9]	30.6 [27.5–33.7]	15.6 [13.2–18.0]	0.9 [0.3–1.5]	3.7 [2.5–5.0]	6.3 [4.7–7.9]
School locality						
urban (*n* = 1831)	61.2 [59.0–63.4]	27.9 [25.9–30.0]	16.3 [14.6–18.0]	0.6 [0.3–1.0]	4.7 [3.7–5.7]	6.2 [5.1–7.3]
semi-rural (n = 873)	61.7 [58.5–64.9]	30.6 [27.5–33.7]	15.6 [13.2–18.0]	0.9 [0.3–1.5]	3.7 [2.5–5.0]	6.3 [4.7–7.9]
rural (*n* = 1562)	61.3 [58.9–63.7]	26.7 [24.5–28.9]	18.2 [16.3–20.1]	0.7 [0.3–1.1]	2.5 [1.7–3.3]	7.7 [6.4–9.0]
Home proximity*						
“mostly close” (*n* = 2519)	60.7 [58.8–62.6]	28.0 [26.3–29.8]	16.8 [15.3–18.3]	0.7 [0.4–1.0]	3.4 [2.7–4.1]	6.7 [5.6–7.8]
“many distant” (*n* = 1747)	62.2 [59.9–64.5]	28.0 [25.9–30.1]	17.0 [15.2–18.8]	0.7 [0.3–1.1]	4.1 [3.2–5.0]	7.0 [5.8–8.2]
**Gender- and age-adjusted prevalences** (% [95% CI])						
Overall (N = 4266)	59.4 [57.9–60.9]	27.3 [26.0–28.6]	16.1 [15.0–17.2]	0.7 [0.5–1.0]	3.5 [3.0–4.1]	6.6 [5.9–7.4]

*CI* confidence interval, *TTH* tension-type headache, *pMOH* probable medication-overuse headache, *d/m* days/month, *UdH* undifferentiated headache; *see text or Table 1 for explanation. Odds ratios for associations with demographic variables are in Table 4, and adjusted odds ratios in Table 5

Because of the high prevalence of migraine, we looked at responses driving this diagnosis. Nausea and vomiting, both specific to migraine, were reported by 48.8% and 14.5% respectively of participants with headache.

### Demographic associations

Headache overall, and all headache types except UdH, were more prevalent among females than males (Tables 2, 3 and 4). All headache types were more prevalent among adolescents than children (Tables 2, 3 and 4). This was true of UdH, although this disorder represented a larger proportion of all reported headache in children (13.0%) than in adolescents (10.0%; *p* = 0.0136 [chi-squared]).

**Table 3:** Bivariate analyses of associations of headache types with demographic variables (*N* = 4266)

Variable	Migraine	Tension-type headache	All headache on ≥ 15 d/m	Undifferentiated headache
	Odds ratio [95% CI]			
Gender				
male (n = 2107)	reference	reference	reference	reference
female (n = 2159)	**1.4** [1.2–1.6]^**3**^	1.2 [0.99–1.4]	**2.0** [1.45–2.7]^**3**^	0.95 [0.75–1.2]
Age group (years)				
6–11 (n = 2241)	reference	reference	reference	reference
12–17 (n = 2025)	**1.9** [1.6–2.1]^**3**^	**2.9** [2.4–3.4]^**3**^	**3.0** [2.2–4.2]^**3**^	**1.5** [1.15–1.9]^**2**^
Pupils’ home-income*				
“higher” (n = 1378)	**0.8** [0.6–0.9]^**2**^	**1.3** [1.03–1.6]^**1**^	0.9 [0.6–1.3]	1.3 [0.9–1.8]
“middle” (n = 2015)	0.9 [0.8–1.1]	1.0 [0.8–1.3]	1.0 [0.7–1.4]	1.0 [0.7–1.35]
“lower” (n = 873)	reference	reference	reference	reference
School locality				
urban (n = 1831)	1.0 [0.9–1.1]	0.9 [0.8–1.1]	**1.5** [1.1–2.0]^**1**^	0.85 [0.7–1.1]
semi-rural or rural (*n* = 2435)	reference	reference	reference	reference

*d/m* days/month; *see text for explanation; significant values are emboldened (^1^p < 0.05; ^2^p < 0.01; ^3^p < 0.001)

**Table 4:** Binary logistic regression analysis of associations of headache types with demographic variables (N = 4266)

Variable	Migraine	Tension-type headache	All headache on ≥ 15 d/m	Undifferentiated headache
	Adjusted odds ratio^**†**^ [95% CI]			
Gender				
male (n = 2107)	reference	reference	reference	reference
female (n = 2159)	**1.4** [1.2–1.6]^**3**^	**1.2** [1.02–1.4]^**1**^	**2.0** [1.5–2.8]^**3**^	0.95 [0.7–1.2]
Age group (years)				
6–11 (n = 2241)	reference	reference	reference	reference
12–17 (n = 2025)	**1.9** [1.65–2.2]^**1**^	**2.9** [2.5–3.5]^**3**^	**3.1** [2.2–4.25]^**3**^	**1.5** [1.2–1.9]^**2**^
Pupils’ home income*				
“higher” (n = 1378)	**0.7** [0.5–0.96]^**1**^	**1.7** [1.2–2.5]^**2**^	0.7 [0.4–1.3]	**2.0** [1.2–3.4]^**1**^
“middle” (n = 2015)	0.9 [0.6–1.3]	1.4 [0.9–2.4]	1.0 [0.4–2.7]	1.7 [0.8–3.5]
“lower” (n = 873)	reference	reference	reference	reference
School locality				
urban (n = 1831)	1.1 [0.9–1.3]	**0.8** [0.6–0.97]^**1**^	**1.6** [1.03–2.5]^**1**^	**0.6** [0.4–0.94]^**1**^
semi-rural or rural (n = 2435)	reference	reference	reference	reference

*d/m* days/month; ^**†**^adjusted for all variables listed; *see text for explanation; significant values are emboldened (^1^p < 0.05; ^2^p < 0.01; ^3^p < 0.001)

The influence of income was inconsistent across headache types: overall there was no difference (Table 2), but migraine was less likely to be reported and TTH and UdH more likely in schools in the higher pupils’ home-income categories (Table 4). Locality also had a variable influence. While, again, there was no difference overall (Table 2), both TTH and UdH, but not migraine, were less likely to be reported in urban schools (Tables 2 and 4). On the other hand, headache on ≥15 days/month was more likely in urban schools (Table 4), but this was not reflected in pMOH (Table 2). Home proximity had no discernible influence (Table 2).

### Headache yesterday (HY)

HY was reported by 679 participants, 26.0% of 2614 with headache (three did not respond to this question) and 15.9% of the total sample. Proportions by gender, age and headache type (of those with headache or the headache type in question) are shown in Table 5. Females (30.3%) reported HY substantially more than males (20.7%; *p* < 0.001), but adolescents (27.0%) only marginally more than children (24.3%; *p* > 0.05). Of the episodic headache types, migraine was most associated with HY, and UdH least, in line, relatively, with their reported headache frequencies (Table 5). Headache on ≥15 days/month, including pMOH, was, proportionately, by far the greatest contributor to HY (≥70%), which was expected since, by definition in these disorders, it must be > 50%.

**Table 5:** Proportions of those with headache reporting headache yesterday, overall and by age, gender and headache type, and predicted proportions, overall and by headache type (*N* = 2614)

Headache type	Headache yesterday				
	Reported proportion n (%)	Predicted proportion			
		Mean reported headache frequency		Predicted headache yesterday (%)	
		days in last week (F7)	days in last 4 weeks (F28)	calculated as F7/7	calculated as F28/28
Any headache (N = 2614)*	679 (26.0)	1.5 ± 1.6	4.1 ± 5.1	21.4	14.6
male (*n* = 1179) female (*n* = 1435)	244 (20.7) 435 (30.3)	not calculated			
6–11 years (*n* = 980) 12–17 years (*n* = 1634)	238 (24.3) 441 (27.0)				
Migraine (*n* = 1192)*	320 (26.8)	1.5 ± 1.5	3.5 ± 3.2	21.4	12.5
Tension-type headache (*n* = 719)	145 (20.2)	1.1 ± 1.4	2.9 ± 3.0	15.7	10.4
Probable medication-overuse headache (*n* = 30)	21 (70.0)	5.0 ± 1.8	20.3 ± 4.3	71.4	72.5
Other headache on ≥15 days/month (*n* = 157)	111 (70.7)	3.9 ± 1.8	18.5 ± 4.3	55.7	66.1
Undifferentiated headache (*n* = 290)	39 (13.4)	0.6 ± 1.0	1.7 ± 2.2	8.6	6.1

*three participants did not respond to this question

Table 5 also shows mean reported headache frequencies based on the two related questions (see Methods). For all episodic headaches, HY was reported more commonly than predicted, more so when prediction was based on the 4-week enquiry. For pMOH, HY was closely matched by both predictions; for other headache on ≥15 days/month, predictions were reasonably approximated.

## Discussion

This was the first enquiry of its type into child and adolescent headache in Western Pacific Region. As similar studies have found in European [13, 14] and African Regions [15], headache had been experienced by most of these young participants (reported lifetime prevalence 81.0%). An estimated 59.4% had an active headache disorder, indicated by headache in the preceding year. Migraine (27.3%) was the most reported type, with TTH (16.1%) following and UdH (6.6%) some way behind. Disorders characterised by headache on ≥15 days/month were far from uncommon (4.2%), although, judged from reported consumption of acute medication, only one sixth of these (0.7%) were pMOH.

We were struck by the high prevalence of migraine, although this finding was not very different from those of other similar studies (see below). Nausea, specific to migraine and a driver of this diagnosis rather than TTH, was reported by almost half (48.8%) of those with any headache, explaining the finding. Special words exist for this symptom in Mongolian language (*dotor muukhairakh*), which are expected to be well understood even by young people.

Females reported more headache of all types except UdH. Adolescents reported substantially more headache of all types including UdH. These associations were mostly as expected, the exceptions for UdH being easily albeit speculatively explained. UdH is understood to represent expressions of migraine or TTH by the immature brain [13], and therefore, by this nature, has an inverse relationship with differentiated headache types as the latter develop. It is not problematic that UdH in females trended in the opposite direction to other headaches. By the same token, the prevalence of UdH as a proportion of all reported headache is expected to decline with increasing age, as was seen in Turkey [13], Lithuania [14] and Austria [22], and as it did here, from 13.0% in children to 10.0% in adolescents.

There were no other associations of interest, except, perhaps, that headache on ≥15 days/month was more likely in urban schools (AOR: 1.6), a finding not reflected in pMOH. Numbers with pMOH were small, so not too much should be made of this. As a general rule, pMOH has two principal drivers. The first is the background prevalence of the antecedent headaches (migraine and TTH), which, although TTH was less prevalent in urban schools, was probably not a factor here. The second is availability of medication, which is usually easier in urban areas. On the other hand, easier access to health care, more usual in urban areas, discourages self-(mis)treatment, or, more probably in this case, (mis)treatment by parents.

It is more interesting to reflect on the overall high prevalence of headache on ≥15 days/month, apparently affecting one participant in every 25 (4.2%). This is a high proportion. Most (70%) of these participants reported HY, giving support to the estimate. We have adult data for comparison from a population-based study in Mongolia, also supported by LTB [12]. Most telling in this study was a prevalence of headache on ≥15 days/month of 10.7%. It appears that the group of disorders this represents, common already in children, becomes more so in adults – an issue of serious public-health concern that calls for investigation (through clinical studies) into what these cases are. Most of the increase from childhood to adulthood is in pMOH, affecting 5.7% of adults (again, incidentally, more of a rural problem) [12].

For further comparison, the gender-adjusted prevalence in adults of all headache in the same study (calculated from the data published) was a not dissimilar 63.7% [12]. Age- and gender-adjusted prevalences in adults were 23.1% for migraine, a small reduction from 27.3%, and 29.1% for TTH, a substantial increase from 16.1%. Of course, there was no UdH in adults. It is easy to speculate that UdH observed in the young participants of the current study is destined to differentiate mostly into TTH, but longitudinal studies are needed to establish this. Two similar studies from Ethiopia, one adult [23] and one in children and adolescents [15], suggested not only that not all childhood headache survives into adulthood, but also what may appear as migraine in children may not persist as migraine in adults. This may be the case in Mongolia, but complicated by the increase with age of headache on ≥15 days/month and, especially, pMOH.

We noted above that HY was reported by 70% of those with headache on ≥15 days/month. This was supported by 1-day prevalence estimates based on reported mean frequency. For the episodic headaches this was not the case. While reported headache days in the preceding one and four weeks were perfectly feasible for migraine, TTH and UdH, the 1-day prevalence estimates that these gave rise to predicted less HY for each of these than was actually reported (by factors of 1.25–1.5 and about 2 for the 1-week and 4-week estimates respectively). Which, it might be asked, are correct? The same question arose in Ethiopia, where the disparities were more extreme [15]. HY reporting should be free from recall error that might, increasingly with longer recall periods, influence retrospective estimates over time. Our findings were consistent with this, but then we must believe that 15.9% (one in six) of these young people had headache on the day in question (and, presumably, a similar proportion on any other day). Were they recalling mild headaches yesterday, more likely to be forgotten over time? The numbers for UdH, by definition a mild headache, might suggest this. We do not present intensity here, but the reported means over the preceding four weeks were 1.7 ± 0.6 for HY against 2.0 ± 0.6 for all headache. So this might have been the case, although this was a small difference on a rather insensitive scale of 1–3.

Comparisons can also be made with children and adolescents in Turkey [13], Lithuania [14] and Ethiopia [15] and with adolescents (10–18 years) in Austria [22], all in studies using the same questionnaire. To facilitate these, we have set out the data in Table 6. These other studies are in close agreement between themselves regarding all headache (72.8–76.6%), significantly higher in all cases than the 59.4% in Mongolia. This difference, however, is entirely accounted for by the children (43.8%): in adolescents, prevalence of all headache was 80.7%. For migraine, Mongolia is at the upper end of the range, except for Ethiopia, which is an outlier among these studies. The clear difference, among the episodic headaches, is in UdH, significantly lower in Mongolia than in all other countries studied. A very possible explanation is reporting bias in respect of a non-troublesome headache, given that UdH by definition is mild and short-lasting, but the truth in this regard is unknown.

**Table 6:** 1-year prevalences (% [95% CI]) of all headache, migraine, tension-type headache and undifferentiated headache in this and other studies using the same questionnaire

	All headache	Migraine	TTH	UdH
Mongolia (all ages) (*N* = 4266)^1^	59.4 [57.9–60.9]	27.3 [26.0–28.6]	16.1 [15.0–17.2]	6.6 [5.9–7.4]
Mongolia (age range 12–17 years) (*N* = 2025)^1^	80.7 [79.0–82.4]	34.6 [32.5–36.7]	24.3 [22.4–26.2]	8.0 (6.8–9.1]
Turkey (*N* = 7068) [13]^1^	73.7 [59.8–62.8]	26.7 [26.7–29.4]	12.9 [15.8–18.0]	29.2 [6.0–7.6]
Lithuania (*N* = 2505) [14]^1^	76.6 [74.9–78.3]	21.4 [19.8–23.0]	25.6 [23.9–27.3]	24.0 [22.3–25.7]
Ethiopia (*N* = 2344) [15]^1^	72.8 [71.0–74.6]	38.6 [36.6–40.6]	19.9 [18.3–21.5]	12.3 [11.0–13.6]
Austria (age range 10–18 years) (*N* = 3386) [22]^2^	75.7 [74.3–77.1]	24.2 [22.8–25.6]	21.6 [20.2–23.0]	26.1 [24.6–27.6]

*TTH* tension-type headache, *UdH* undifferentiated headache; ^1^ age- and gender-adjusted; ^2^ crude (observed) values

The strengths of this study lie in its adequate sample size, the validity of schools-based sampling in this country, the high participation proportion, and the tested and validated methodology [11]. The principal limitation was uncertainty over the quality of responses from children, and therefore of case ascertainment in this group, but this is an inherent and unavoidable problem. We did our best to mitigate it through mediated enquiry. With regard to UdH, diagnosis is dependent on assessment of headache intensity, always highly subjective, and on estimates of attack duration. Children in particular do not have a good sense of time.

## Conclusions

At least in adolescents, headache is no less common than it is in adults in Mongolia, nor in this age group than in other countries. The findings of this study are available to inform educational and health policies in Mongolia. With no similar study yet from elsewhere in the whole of Western Pacific Region, they also make an important contribution to LTB’s global enquiry into child and adolescent headache, a major fact-finding component of the Global Campaign against Headache [7, 8].

## Data Availability

The data are held on file at University of Mersin. Once analysis and publications are completed, they will be freely available for non-commercial purposes to any person requesting access in accordance with the general policy of the Global Campaign against Headache.

## Abbreviations

AOR: adjusted odds ratio
CI: confidence interval
d/m: days per month
GBD: Global Burden of Disease (Study)
HARDSHIP: Headache-Attributed Restriction, Disability, Social Handicap and Impaired Participation (questionnaire)
HY: headache yesterday
ICHD: International Classification of Headache Disorders
IHS: International Headache Society
LTB: *Lifting The Burden*
MNUMS: Mongolian National University of Medical Sciences
MOH: medication-overuse headache
OR: odds ratio
pMOH: probable MOH
SD: standard devation
TTH: tension-type headache
UdH: undifferentiated headache
